# An in-depth review on utilizing ultrasound biomicroscopy for assessing the iridocorneal angle and ciliary body in canines

**DOI:** 10.3389/fvets.2025.1501405

**Published:** 2025-02-26

**Authors:** Donghee Kim, Hyun Kwon, Jiyi Hwang, Ji Seung Jung, Kyung-Mee Park

**Affiliations:** Laboratory of Veterinary Surgery and Ophthalmology, College of Veterinary Medicine, Chungbuk National University, Cheongju, Republic of Korea

**Keywords:** ultrasound biomicroscopy, iridocorneal angle, ciliary body, ciliary cleft, canine

## Abstract

In this review, we explore the transformative role of Ultrasound Biomicroscopy (UBM) in veterinary ophthalmology, focusing on its utility in evaluating the iridocorneal angle and ciliary body in dogs. We begin by outlining UBM’s foundational principles, providing a holistic understanding of its operational mechanics. This is followed by an exploration of the techniques and considerations for optimal UBM imaging, including the use of topical anesthesia, probe positioning, and maintaining a controlled measurement environment. A major section is dedicated to the detailed anatomy of the anterior segment, emphasizing the iridocorneal angle and ciliary body in controlling aqueous humor dynamics within canine and feline eyes. By comparing anatomical structures in humans and animals, we highlight the need for distinct parameters in veterinary medicine. The review also analyzes the parameters obtainable via UBM, emphasizing its potential in monitoring drug-induced ocular changes, gaging post-cataract surgical outcomes, and observing inter-species variations. We conclude by encapsulating the current state of research, addressing existing challenges, and suggesting future research avenues. This synthesis underscores the pivotal role of UBM in advancing veterinary ophthalmic diagnostics and research.

## 1 Introduction

### 1.1 Introduction to ultrasound biomicroscopy

The development of Ultrasound Biomicroscopy (UBM) began with the work of Pavlin et al. in the early 1990s, introducing high-magnification imaging of anterior segment structures (1, 2). UBM provides detailed visualization of the eye’s structures using high-frequency (35–100 MHz) ultrasound technology (2–4). The ultrasound waves, reflected by ocular tissues, are converted into visual representations, allowing for imaging of the anterior chamber, iridocorneal angle (ICA), and ciliary body with axial and lateral resolutions of approximately 0.2 mm and 0.5 mm, respectively (2). UBM is particularly effective in visualizing anterior segments of the eye that are not easily seen with conventional imaging techniques. Its resolution and depth of penetration, achieved with operational frequencies between 35 and 100 MHz, allow for detailed visualization of ocular structures from the corneal endothelium to the posterior lens capsule (2–4).

### 1.2 Comparison of UBM and alternate imaging techniques

Optical Coherence Tomography (OCT) is widely used for imaging the retina and anterior segment, employing infrared ray measurements to produce high-resolution images (5–7). However, OCT encounters limitations when imaging non-transparent or dense structures, such as thick iris tissue and the pigment epithelium (3). In contrast, UBM’s ability to penetrate these barriers allows for visualization of deeper anatomical structures and lesions, even in cases where the cornea is opaque or where there is the presence of hyphema (3, 8). This characteristic makes UBM particularly effective in situations where OCT’s performance may be limited.

Nevertheless, while UBM is advantageous for visualizing deeper or denser anterior structures, OCT excels in providing high-resolution images of transparent structures and the retina (2, 4, 6). Each modality offers unique strengths and limitations, making them complementary tools rather than direct competitors. The choice of imaging technique should be guided by the specific clinical need, with UBM and OCT often used together to provide a comprehensive view of ocular structures.

In veterinary medicine, Sim et al. utilized spectral domain OCT (SD-OCT) to study the ICA (8). However, many commercial SD-OCT devices have limitations due to their shorter wavelengths (800–880 nm), which restrict tissue penetration and complicate visualization of areas such as the angle recess and ciliary cleft (CC) in dogs (9). Additionally, the light from SD-OCT can be attenuated or absorbed by pigmented tissues, making it challenging to evaluate through pigmented corneas and posterior iris structures. In contrast, UBM is more effective in these situations, offering valuable insights into structures such as the iridociliary complex, zonular abnormalities, and underlying iris masses. Its ability to provide dynamic imaging enhances its utility, especially for evaluating the relationships between ocular structures, such as during angle assessments or post-surgical lens fit evaluations.

High-resolution ultrasound (HRUS), like UBM, is an advanced ultrasound imaging technique used for medical diagnostics (4). HRUS operates at lower frequencies (20–50 MHz) compared to UBM, offering slightly lower resolution but greater depth of penetration (4, 10). For this reason, UBM is primarily used for imaging the anterior segment of the eye. Its ability to provide detailed imaging of structures behind the iris, such as the ciliary body and lens, makes it particularly useful in glaucoma assessment and the diagnosis of anterior segment pathologies (10). Despite UBM offering more detailed imaging, higher resolution does not necessarily correlate with greater clinical utility in every setting. HRUS can provide adequate visualization in many clinical contexts where UBM’s higher resolution is not required.

Moreover, while UBM requires a water bath or gel interface and may necessitate more specialized equipment, HRUS is generally easier to perform and more widely available in clinical settings (2, 11). The choice between HRUS and UBM depends on the specific anatomical region and diagnostic requirements. UBM provides superior detailed imaging for anterior eye segments, whereas HRUS offers a balance of high resolution and adaptability for various clinical applications across the body (Table 1).

**Table 1:** Comparative summary of ocular imaging modalities: UBM, OCT, and HRUS.

Parameter	UBM (ultrasound biomicroscopy)	OCT (optical coherence tomography)	HRUS (high-resolution ultrasound)
Imaging principle	High-frequency ultrasound	Interferometric imaging using near-infrared light	Moderate-frequency ultrasound
Frequency	35–100 MHz	Not applicable (light-based) (~840 nm or 1,310 nm)	20–50 MHz
Resolution	~20–50 μm	~5–10 μm	~30–100 μm
Resolution characteristics	Optimized for detailed anterior segment imaging	Ideal for visualizing transparent tissues (e.g., retina, cornea)	Suitable for various clinical applications
Penetration depth	~4–5 mm	~2–3 mm	Up to 1–2 cm
Advantages	Excellent for imaging opaque or pigmented anterior structures Enables dynamic assessments	Non-contact and rapid acquisition High resolution for transparent structures	Widely available and easy to perform Balances resolution and penetration for various applications
Limitations	Requires a water/gel interface Optimized mainly for the anterior segment	Limited penetration in dense tissues Image quality can be affected by tissue pigmentation	Lower resolution compared to UBM May exhibit ultrasound artifacts
Clinical applications	Anterior segment evaluation (e.g., iridociliary complex, glaucoma assessments, pre−/post-surgical imaging)	Retinal imaging, assessment of transparent ocular structures, and anatomical studies	Supplemental imaging in ocular diagnostics and broader clinical applications

## 2 Techniques and considerations for UBM imaging

### 2.1 Positioning and technique variations with traditional immersion method and the sterile balloon method

UBM utilizes high-frequency ultrasound to capture detailed images, requiring a water-based coupling medium to reduce reflections between the probe and the eye (12, 13). There are two primary methods for containing the coupling medium: the immersion method and the sterile balloon (ClearScan^®^) method (14). The choice of method influences factors such as positioning, sedation, and procedural requirements (Table 2).

**Table 2:** Comparison of traditional immersion and sterile balloon methods for UBM imaging.

Parameter	Immersion method	Sterile balloon method
Patient positioning	Dorsal recumbency with head stabilization	Sternal recumbency with manual restraint
Coupling medium	Water-based medium contained in a tapered cupule	Medium enclosed within a sterile balloon
Sedation anesthesia	Sedation or anesthesia	Without sedation
Pressure compression	Continuous pressure needed; risk of iridocorneal angle distortion	Minimal compression; reduced risk of anatomical distortion
Image quality	Typically yields clearer images with stable coupling	Slightly reduced clarity compared to immersion
Procedural simplicity	More complex due to sedation and pressure management	Simpler execution with fewer procedural requirements

In the immersion method, the patient’s head is stabilized in dorsal recumbency using a hollow cushion. A tapered cupule is inserted into the palpebral fissure, acting as both a speculum and a watertight chamber for the interface liquid, such as lactated Ringer’s solution. This method typically requires the patient to remain still, often necessitating anesthesia to maintain the water-filled state (15, 16). In contrast, the sterile balloon method allows examination with manual restraint in sternal recumbency, with the eyelids manually held open (10, 17–19).

According to the author’s experience, the immersion method tends to yield clearer images. However, this technique is challenging in veterinary ophthalmology. Specifically, it requires the patient to be sedated or anesthetized, which is not always ideal in a clinical setting. Moreover, to prevent the water from leaking out of the chamber, continuous pressure must be applied to the eye. This compression can lead to distortion of the iridocorneal angle. In contrast, the balloon method offers the advantage of being performed without sedation, is simpler to execute, and minimizes the distortion associated with compression.

### 2.2 Topical anesthesia and probe positioning

Both methods typically involve the topical application of anesthetics, such as 0.5% proparacaine hydrochloride or 0.4% oxybuprocaine hydrochloride, to facilitate probe positioning (19). This allows for easy examination of the superior and temporal quadrants of the eye (10 to 3 o’clock positions), which are the most accessible areas in dogs (10, 17). The probe is positioned perpendicular to the globe, with the scan plane aligned perpendicular to the limbus. For the sterile balloon method, hydroxymethylcellulose is applied to protect the cornea and sclera from hypoallergenic ultrasound gel (17).

### 2.3 Consistency in measurement environment

Maintaining consistency in lighting conditions, pupil dilation status, and the use of pharmacological agents that affect these parameters is essential for accurate UBM imaging. Rose et al. demonstrated decrease in ICA parameters after pupil dilation in normal eyes without cataracts (20). Additionally, the use of topical agents can induce contraction or relaxation of the ciliary muscle, leading to alterations in the configuration of the ciliary body (15, 21, 22). Therefore, controlling experimental conditions is crucial. To achieve consistency, some studies monitor and maintain the background room illuminance in lux units and regulate the degree of pupil dilation affected by lighting (23). However, stress or excitement in animals visiting the clinic can cause the pupil to assume a midrange or dilated position, which may influence UBM measurement results (24). For this reason, some studies standardize conditions using 1% tropicamide to ensure uniformity in pupil dilation (25, 26).

Nevertheless, this approach also has its drawbacks. There is a possibility that individual responses to the mydriatic agent may distort the results. For example, while 0.5% tropicamide can be used to consistently maintain a dilated state, the response of the ciliary body can vary among subjects. In other words, although tropicamide is known to relax the ciliary body, it does not do so uniformly in all individuals (27). Therefore, the author believes that the best way to prevent distortion is to avoid using pharmacological agents altogether and to capture images under consistent lighting conditions when the patient is calm.

## 3 Role of ultrasound biomicroscopy in anterior segment evaluation

### 3.1 Comprehensive examination of the iridocorneal angle and the ciliary body anatomy

**Figure 1:**
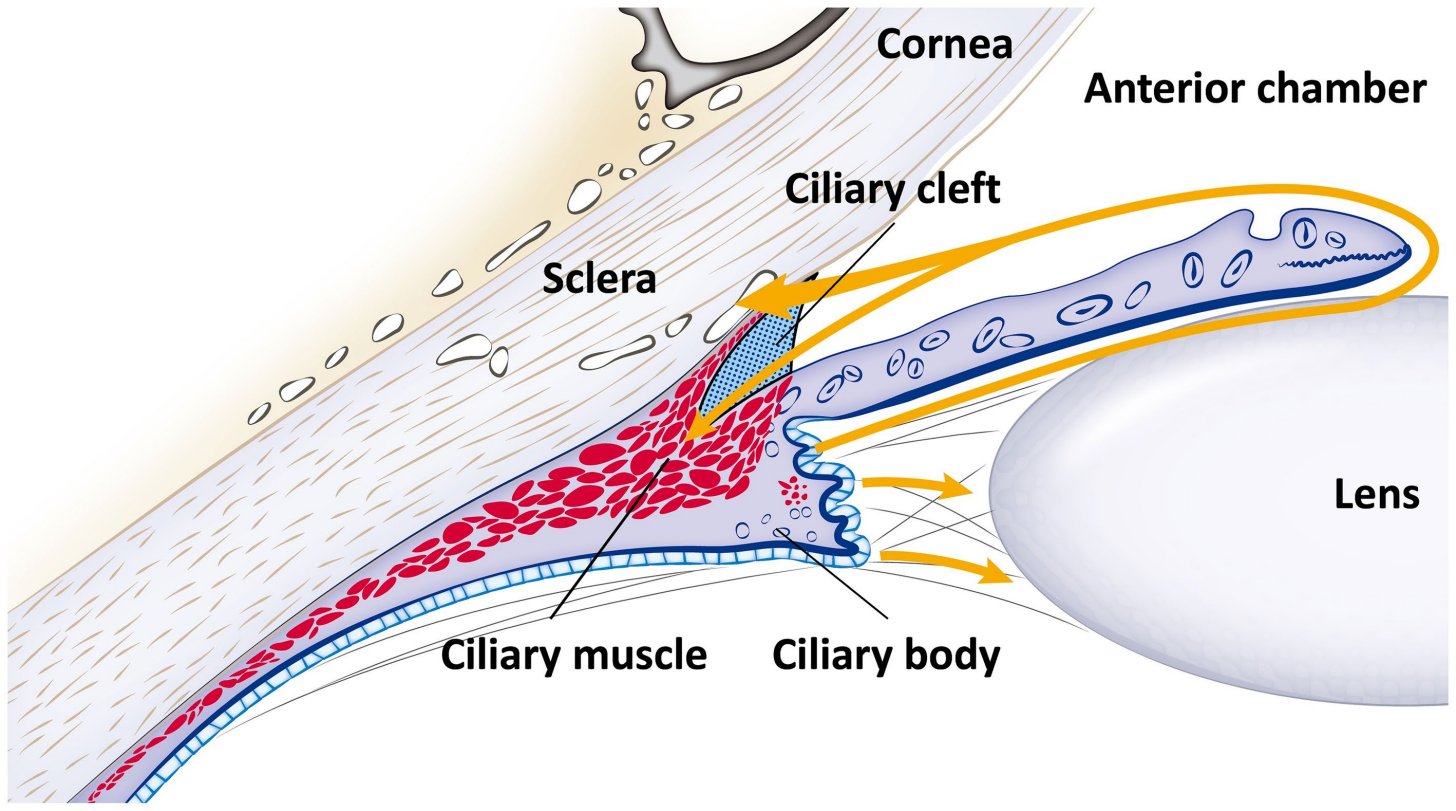
Schematic diagram of the iridocorneal angle and ciliary body anatomy, with aqueous humor flow. This schematic representation details the anatomy of the iridocorneal angle and the ciliary body, along with the pathway of aqueous humor flow (indicated by the yellow line with an arrow). Aqueous humor is produced by the ciliary body and flows from the posterior chamber, through the pupil, into the anterior chamber. Subsequently, it exits via the ciliary cleft into the radial collector channels and is then absorbed into the episcleral veins, following the conventional pathway. Additionally, a portion of the aqueous humor diverges into the longitudinal muscle fibers of the ciliary body, illustrating the unconventional pathway.

The ICA and ciliary body are key ocular structures involved in the regulation of aqueous humor (AH) flow in dogs. The ICA functions as a crucial site for AH outflow, while the ciliary body is responsible for AH production (28, 29). Together, these structures play essential roles in both the formation and regulation of AH dynamics (Figure 1).

In the conventional outflow pathway, AH exits the eye through the corneoscleral trabecular meshwork (TM). The ICA forms a peripheral portion of the anterior chamber, where the cornea, sclera, and the base of the iris meet (16, 30). The ICA is composed of a reticular network of connective tissue beams, known as trabeculae, which are lined either partially or entirely by a single layer of cells (16, 31, 32).

Using clinical examination techniques such as gonioscopic lenses, the distinctive features of the ICA can be observed, including slender strands of uveal tissue and pectinate ligaments (PLs), which connect parts of the iris to the peripheral cornea (16, 33–36). In ICAs characterized by stout PLs, the anterior chamber communicates freely with the ICA through pores that lead into a network of small channels (29). These channels are surrounded by cords of densely packed collagen. Further posteriorly, the PLs interconnect with the anterior beams of the TM, illustrating the complex and finely tuned structure of the outflow pathway (37).

The CC, which is not present in humans but is a characteristic structure in dogs, is a vital triangular space located posterior to the ICA. Functioning as a posterolateral extension of the anterior chamber into the ciliary body, the CC varies in both depth and height. Historically referred to as the cilioscleral sinus, this term has been replaced with CC, as it neither separates the ciliary body from the sclera nor is part of the ciliary venous sinus (30, 38). This virtual space extends beyond the PLs into the posterior ciliary body, forming a triangular shape with an anterior base. Anatomically, the boundaries of the CC include the PLs anteriorly, the iris root and anterior pars plicata of the ciliary body internally, the ciliary body matrix and muscle posteriorly, and the sclera externally (28, 39, 40). The CC is characterized by wide spaces filled with AH and interspersed with cell-lined cords of connective tissue (40). The CC plays a critical role as the container for the uveal trabecular meshwork, a key component in regulating intraocular pressure (IOP) and maintaining overall eye health in dogs.

The ciliary body muscle in a dog’s eye is a smooth, nonstriated muscle located primarily in the anterior two-thirds of the ciliary body stroma and is divided into three distinct parts. The outer longitudinal portion, known as Brücke’s muscle, is the most external and closest to the sclera (41). It forms a V-shaped structure that constitutes the bi-leaflet of the CC, with carnivorous species exhibiting a similar bi-leaflet configuration but with variations in muscle fiber orientation (38, 42). Contraction of this portion can reduce the space between muscle bundles, decreasing aqueous outflow (43). The middle oblique or reticular portion attaches to collagenous structures near the ciliary processes, while the inner circular part, referred to as Müller’s muscle, acts as a sphincter near the major arterial circle of the iris (44). This inner portion influences lens refraction by pulling the ciliary processes and body forward and inward, relaxing the lenticular zonules and altering the lens shape (45–47). The different muscle fibers exhibit ultrastructural and histochemical differences, with the longitudinal portion primarily involved in regulating outflow and the circular portion related to accommodation (48).

### 3.2 Comparative anatomy of iridocorneal angle and ciliary body in humans and dogs

**Figure 2:**
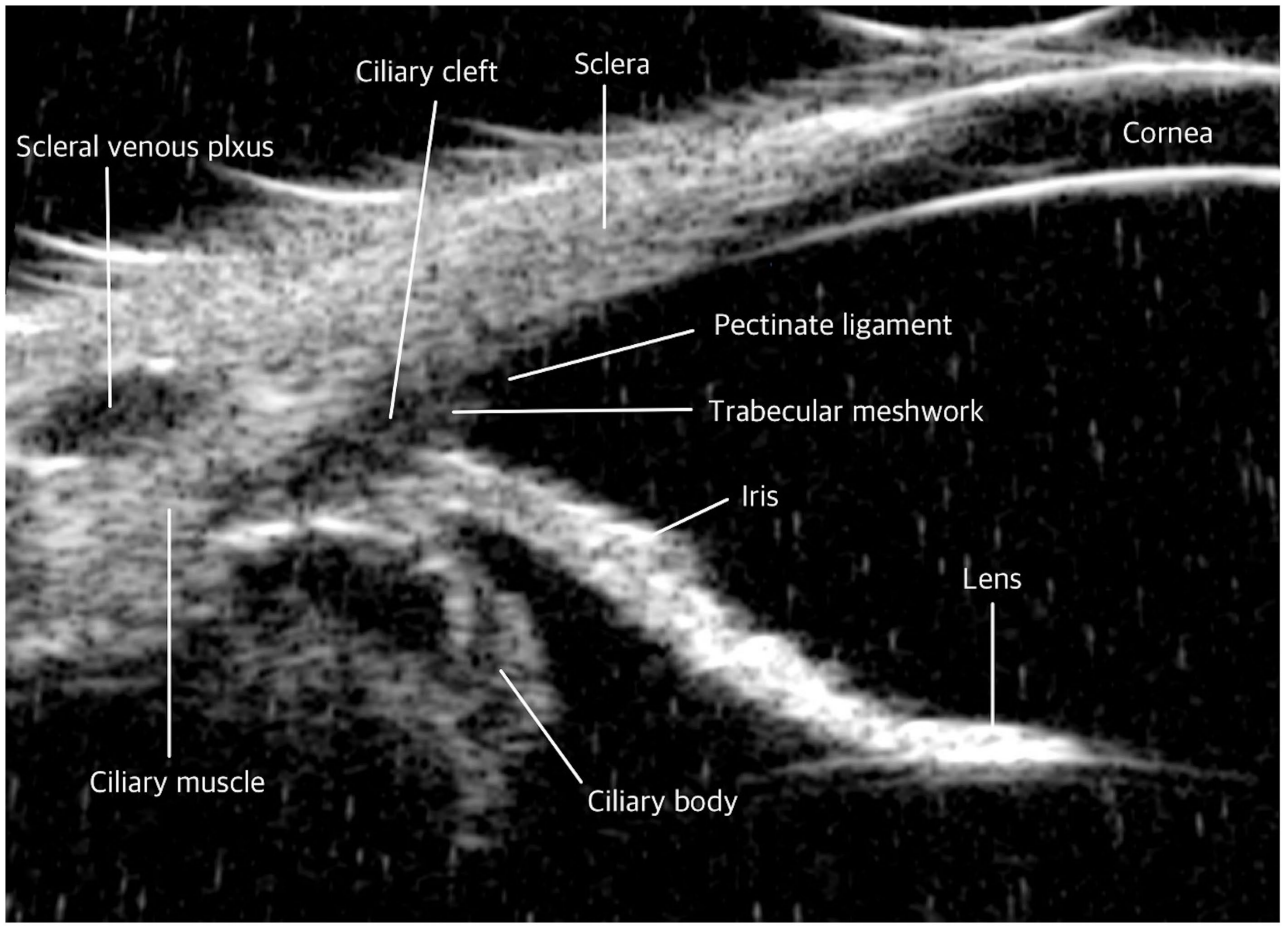
Comparative ultrasound biomicroscopy (UBM) images of the iridocorneal angle in dog. Iridocorneal angle: In contrast to humans, dogs do not possess a scleral spur. The trabecular meshwork in dogs is uniquely structured; it is exposed beyond the sclera and situated within the recess of the ciliary cleft.

There are anatomical differences in the ICA between humans and dogs (49, 50). In humans, the scleral spur is present as a fibrous ring projecting from the inner aspect of the anterior sclera in a meridional section. The scleral spur is attached anteriorly to the TM and posteriorly to the sclera and the longitudinal portion of the ciliary muscle (51, 52). It serves as an important landmark for determining angle configuration due to its distinctive outline and higher reflectivity compared to the ciliary body, aiding in the identification of the angle during imaging (1, 53). However, this structure is absent in dogs, which can make consistent landmark identification using UBM more challenging. Additionally, in dogs, the TM is located within the recess of the CC, whereas in humans, it is positioned anteriorly relative to the iris root and resides within the scleral sulcus (Figure 2) (28, 40, 48, 54–56). As a result, iris dilation in humans can obstruct access to the TM by causing external and anterior displacement of the iris root (57, 58). In contrast, the location of the TM in dogs makes it less susceptible to such obstructions, ensuring that iris movement has a minimal effect on AH outflow. Therefore, a different approach may be necessary when using UBM in humans compared to dogs.

The ciliary body in humans displays distinct anatomical differences compared to other species. The human ciliary body musculature is highly developed, comprising three distinct components: radial, meridional, and circular fibers. These fibers form a prominent anterior pyramidal structure, which provides a stable and robust base for iris attachment (59). The development of the circular fibers within this muscle facilitates the anterior inward movement of the ciliary body (60). In humans, the anterior segment of the ciliary body muscle has largely taken over the functions of the CC, which is nearly absent, and the PLs, which persist only as sparse iridal processes (40). In contrast, canine anatomy is characterized by a predominance of meridional (or longitudinal) fibers in the ciliary body muscle, with fewer circular fibers compared to humans (61, 62). Therefore, in dogs, it is believed that the anterior inward movement of the ciliary body is primarily regulated by meridional fibers rather than circular fibers (Table 3).

**Table 3:** Comparative anatomy of the iridocorneal angle and ciliary body in humans and animals.

Anatomical region	Parameter	Humans	Dogs
Iridocorneal angle	Scleral spur	Present as a fibrous ring; serves as a key landmark	Absent
	Trabecular meshwork	Located anterior to the iris root in the scleral sulcus	Located within the recess of the ciliary cleft
	Iris dilation effect	Iris dilation can obstruct TM access	Minimal impact due to the recessed TM position
Ciliary body	Muscle composition	Radial, meridional, circular fibers	Predominantly meridional fibers
	Structural features	Prominent pyramidal structure; ciliary cleft nearly absent	Less developed CC; anterior movement mainly via meridional fibers

### 3.3 Comparison of histological and UBM images

**Figure 3:**
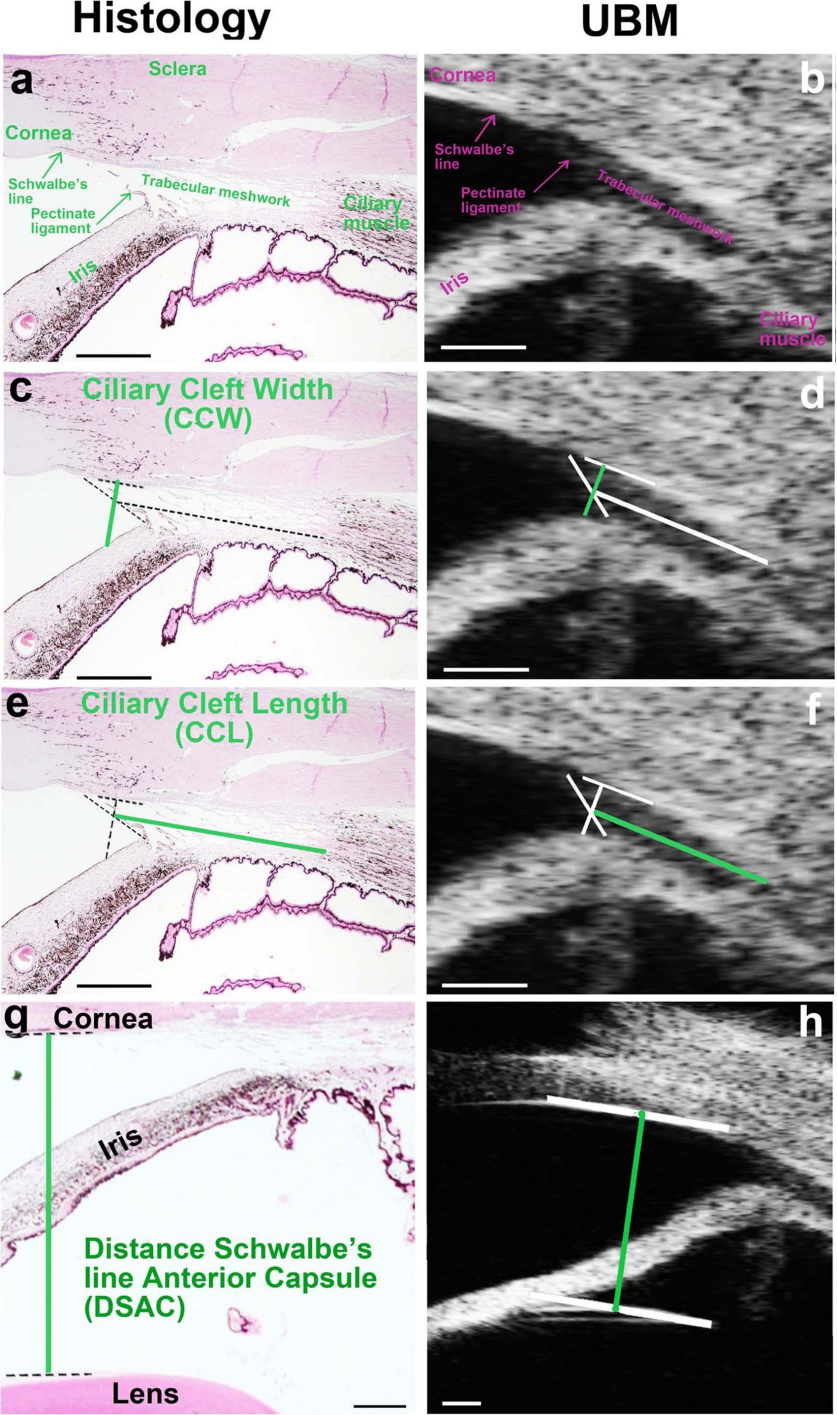
Comparison of histological and UBM images of the iridocorneal angle (ICA) in dogs. **(a, c, e, g)** Histological section of the ICA stained with hematoxylin/eosin, highlighting key anatomical structures including the pectinate ligaments (PLs), trabecular meshwork (TM), and ciliary cleft (CC). **(b, d, f, h)** Corresponding UBM image of the same ICA region, demonstrating the ability of UBM to delineate these structures *in vivo*. The PLs are distinctly visible in both imaging modalities, forming the anterior boundary of the CC. The TM, occupying the CC, is observed in both images, though finer cellular details are more clearly defined in histological sections. Reproduced from Boillot et al. (16), CC BY 4.0.

To validate the accuracy of UBM in visualizing anterior segment structures, histological sections of the ICA were compared with corresponding UBM images obtained from the same subjects. Figure 3 illustrates these comparisons, highlighting key anatomical landmarks observed in both imaging modalities (16).

Histological sections stained with hematoxylin/eosin (Figure 3A) provided a detailed view of ICA structures, including the PLs, TM, and CC. These features were also visualized in UBM images (Figure 3B), demonstrating the capacity of UBM to delineate ICA structures *in vivo*. The PLs were clearly identified in both histological and UBM images, marking the anterior boundary of the CC. The TM, occupying the CC, was similarly observed in both modalities, although the histological sections revealed finer details of its cellular organization, which could not be resolved with UBM.

## 4 Interpreting parameters evaluated via UBM in the assessment of the iridocorneal angle and ciliary body

### 4.1 Iridocorneal angle parameters: ICA, AOD

The ICA and angle-opening distance (AOD) are commonly used parameters in both human ophthalmology and veterinary practices for animals such as dogs (53, 63). The ICA is typically measured by locating the apex of the angle at the junction of the iris, TM, and sclera. From this apex, lines are extended along the inner surface of the sclera and iris to the level of the limbus, allowing for precise measurement (15, 20, 33).

**Figure 4:**
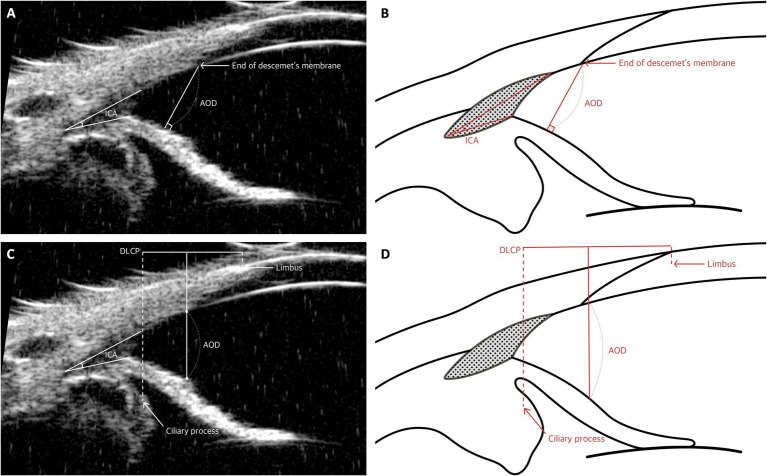
Measurement techniques of the iridocorneal angle (ICA) and angle-opening distance (AOD) utilizing UBM images in dog. **(A,B)** Measurement of ICA and AOD: These panels demonstrate the method for measuring both the iridocorneal angle and the AOD. The apex of the ICA is positioned at the junction where the iris, trabecular meshwork, and sclera meet, with measurement lines extending from this apex along the inner surface of the sclera and iris up to the level of the limbus to determine the angle. Additionally, the AOD is quantified as the distance from the end of Descemet’s membrane to the iris. **(C,D)** Measurement of AOD: These images depict the technique for approximating AOD using the distance from the limbus to the ciliary process (DLCP). A perpendicular line to the DLCP is drawn from a point slightly below the midline, specifically at 0.45 times the DLCP, extending from the sclera to the iris.

For AOD, several distinct methodologies are employed. One method defines the AOD as the distance, measured in micrometers, from the end of Descemet’s membrane to the surface of the iris (Figures 4A,B) (8). Another method involves drawing a 500-μm line from the apex of the ICA to a point on the inner corneoscleral surface, from which a perpendicular line is drawn to the iris, and the length of this line is measured (15). A third method estimates the AOD by using the distance between the limbus and the ciliary process (DLCP). A line perpendicular to the DLCP is drawn from a point located under the midline (0.45 × DLCP, measured from the ciliary process) from the sclera to the iris; this line represents the AOD (Figures 4C,D) (50)

These parameters have been adapted from human ophthalmology to account for the distinct anatomical structures of dogs. In human studies, AOD is frequently measured using the scleral spur as a consistent anatomical landmark, particularly when assessing AOD at 500 μm (AOD500) (64). However, in animals such as dogs and cats, the absence of a consistent landmark like the scleral spur has necessitated the use of alternative reference points, such as the angle recess and Descemet’s membrane (8, 15).

The importance of these parameters is evident in their application for evaluating angle-closure glaucoma (ACG) in humans (64, 65). ACG refers to a group of diseases in which there is either reversible or adhesional closure of the anterior chamber angle, obstructing AH flow through the canal of Schlemm (66). The ICA and AOD parameters are used to assess the proximity of the iris to the scleral spur, which serves as a landmark for the canal of Schlemm (67). However, there is skepticism regarding the relevance of these parameters in dogs, given their anatomical differences from humans (26). The use of the angle recess and Descemet’s membrane as alternative landmarks may not have the same significance in these species, raising debate about the appropriateness and interpretation of these measurements in veterinary ophthalmology (26).

### 4.2 Quantitative analysis of iris configuration using UBM parameters

**Figure 5:**
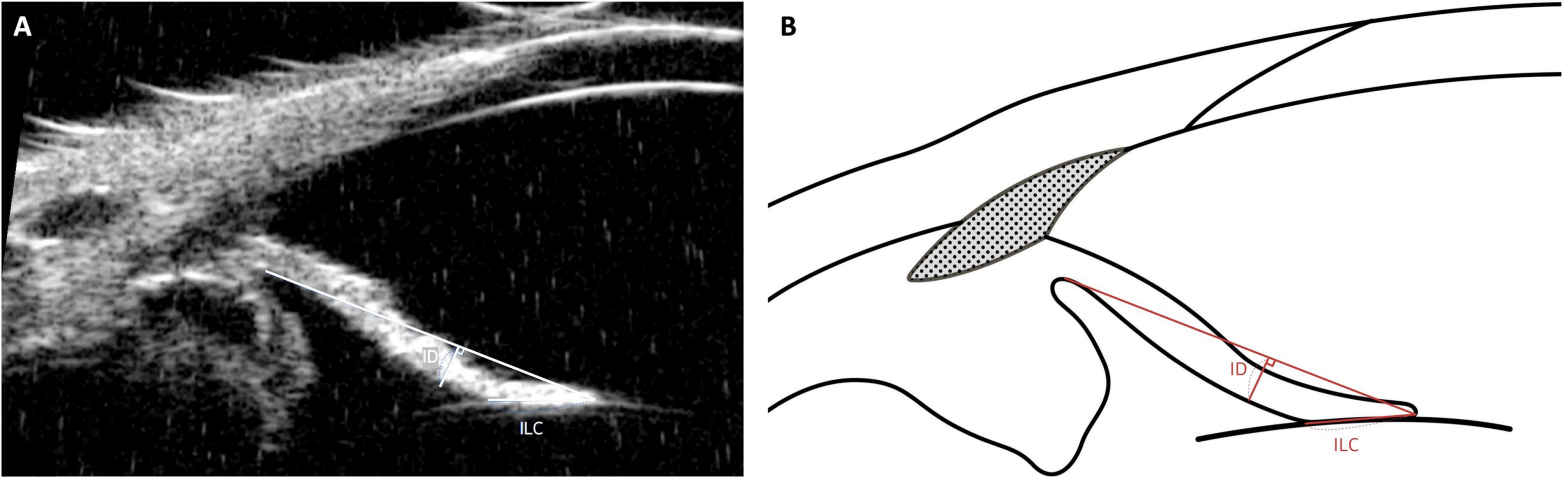
Measurement techniques of iris-lens contact (ILC) and iris deviation (ID) using UBM images in dog. This figure demonstrates the methods used to measure ILC and Iris Deviation (ID). The ILC is assessed by measuring the distance between the innermost and outermost contact points of the iris’s posterior pigmented epithelium with the anterior capsule of the lens. The ID provides insights into the curvature of the iris, measured from the point of iris-lens contact to the furthest edge at the iris root.

The morphological characteristics of the iris play a crucial role in anterior segment dynamics and AH outflow regulation. To quantitatively assess these structural variations, two key parameters, iris-lens contact (ILC) and iris deviation (ID), were evaluated using UBM. These parameters provide insight into anatomical factors that may contribute to primary angle-closure glaucoma (PACG) (Figure 5) (49, 68).

ILC represents the extent of contact between the posterior iris and the anterior lens capsule. An increased ILC value indicates greater contact, which can lead to pupillary block, where aqueous humor accumulation in the posterior chamber causes anterior displacement of the peripheral iris, narrowing the iridocorneal angle. In human ophthalmology, elevated ILC is a well-established risk factor for PACG (69, 70). While a direct correlation in veterinary medicine has yet to be confirmed, comparative studies suggest that breeds predisposed to PACG, such as American Cocker Spaniels, exhibit higher ILC values than breeds with lower glaucoma incidence, such as Beagles (49).

ID quantifies the curvature of the iris by measuring its deviation from a reference baseline. A higher ID value indicates greater anterior bowing of the iris, which can contribute to angle crowding and increased AH outflow resistance. Conversely, a lower ID value suggests a flatter iris profile, typically associated with an open iridocorneal angle and lower risk of AH obstruction (24). Given the role of anterior iris displacement in PACG, ID may serve as a useful supplementary parameter alongside ICA and ciliary cleft width (CCW) for evaluating anatomical predisposition to glaucoma.

While ILC and ID have been extensively studied in human glaucoma research, their clinical relevance in veterinary ophthalmology requires further investigation (71, 72). Establishing normative values for these parameters in different breeds and assessing their changes over time may help clarify their role in PACG pathogenesis. Future research should also explore their potential as early indicators of disease progression and treatment response.

### 4.3 Ciliary cleft parameters: CCW, mid-CCW, CCL, CCA

The CC serves as a common pathway for the outflow of AH, regulating its flow (28). It plays a significant role in various research and clinical applications, such as the analysis of breeds with glaucoma predisposition, the examination of drug responses to glaucoma medications, and studies to identify the causes of post-operative hypertension (POH) or glaucoma before and after cataract surgery, utilizing parameters associated with the CC (15, 16, 18, 19, 23, 73).

**Figure 6:**
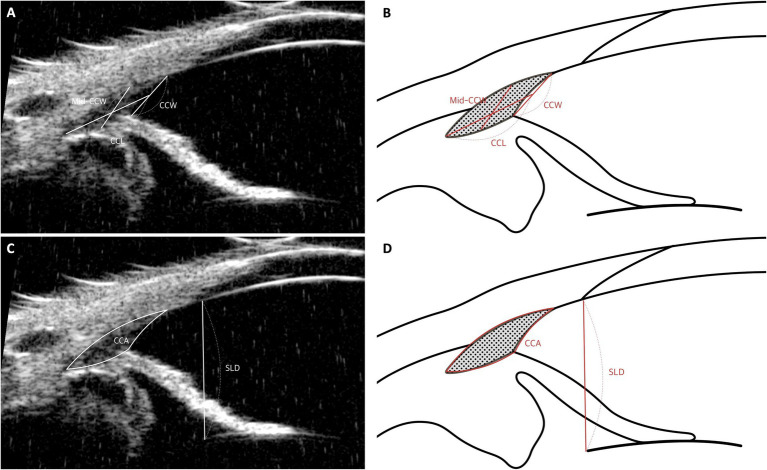
Measurement techniques of ciliary cleft parameters using UBM images in dog. **(A,B)** Measurement of ciliary cleft parameters: The width at the ciliary cleft (CCW) is quantified by the distance from the corneoscleral limbus to the iris root. The width at the mid-point of the cleft (Mid-CCW) is calculated between the inner wall of the sclera and the nearest ciliary process. The length of the ciliary cleft (CCL) spans from the pectinate ligament or the most forward part of the uveal trabecular meshwork to the front of the ciliary body. **(C,D)** Measurement of ciliary cleft area and Schwalbe’s line to lens distance (SLD): The ciliary cleft area (CCA) is determined by outlining the area formed by the CCW and lines delineating the inner scleral boundary from the inner sclera to the angle recess, along with a line marking the upper edge of the iris root from the angle recess to the top of the iris root. Additionally, the distance from Schwalbe’s line, which marks the transition from cornea to sclera, to the anterior lens capsule (SLD) is meticulously measured.

The parameters used to assess the CC include:

1. Ciliary Cleft Width (CCW): This is measured as the distance from the point where the PLs contact the sclera to the point where they meet the iris (Figures 6A,B).
2. Mid-Ciliary Cleft Width (Mid-CCW): This represents the distance between the inner sclera and the ciliary process in the central portion of the CC (Figures 6A,B).
3. Ciliary Cleft Length (CCL): This measurement extends from the angle recess to the midpoint of the PLs (Figures 6A,B).
4. Ciliary Cleft Area (CCA): The CCA is calculated as the area enclosed by the CCW, the lines tracing the inner scleral side of the CC from the inner surface of the sclera to the angle recess, and the line tracing the superior surface of the iris root from the angle recess to the superior surface of the iris root (Figures 6C,D).

Comparing CCW and CCA measured by UBM across dogs can be challenging, as these values may vary due to differences in ocular size, which are often influenced by body size or weight (74). In a study by Kawata et al., dogs were classified into four groups based on body weight, and differences in CCW and CCA were observed between the groups (17). To enable valid comparisons between animals of varying sizes and weights, the CCW and CCA values obtained from UBM images must be adjusted or rectified. In this study, rectification was achieved using the distance from Schwalbe’s line (the boundary between the cornea and sclera) to the anterior lens capsule (SLD) (Figures 6C,D) (17, 23, 75). The researchers employed an optional fixed SLD (OFS), such as the mean SLD of the examined dogs. The formulas for rectified CCW (r-CCW) and rectified CCA (r-CCA) were as follows:


$$r-CCW=CCW\times \left(OFS/SLD\right)$$

$$r-CCA=CCA\times {\left(OFS/SLD\right)}^{2}\text{.}$$

After rectification, no differences were found in r-CCW and r-CCA across the groups (17). These findings highlight the importance of accounting for body size and weight when evaluating CC parameters using UBM in veterinary ophthalmology.

However, there are limitations when comparing different sexes using this rectification method. The underlying assumption in this approach is that there is no sex difference in the SLD. In contrast, our previous study reported that the peripheral-anterior chamber depth corresponding to the SLD is smaller in female dogs (76). Consequently, when applying the rectification method, the rectified values (r-CCW and r-CCA) may be measured as smaller in female dogs compared to male dogs. Therefore, caution is warranted when utilizing this method for inter-sex comparisons.

### 4.4 Ciliary body movement parameters: CPSA, CBAXL

In human medicine, ciliary body movement plays a crucial role in controlling accommodation, and UBM studies have been conducted to investigate this aspect (77–79). Accommodation refers to the eye’s ability to adjust its refractive power, allowing for clear vision of objects at different distances (80). During accommodation, the ciliary muscle contracts, causing the ciliary body to move both forward and inward. This movement reduces tension on the zonule, enabling the lens equator to shift further from the sclera (45, 81).

While the significance of ciliary body movement is generally considered low in dogs, recent studies have linked it to the expansion and contraction of the CC (25, 26). This connection is based on the fact that the CC is enclosed by the inner and outer leaflets of the longitudinal ciliary muscle (38, 42). For example, ciliary body contraction causes the ciliary muscle to move inward and anteriorly, leading to the compression and subsequent contraction of the CC (25).

**Figure 7:**
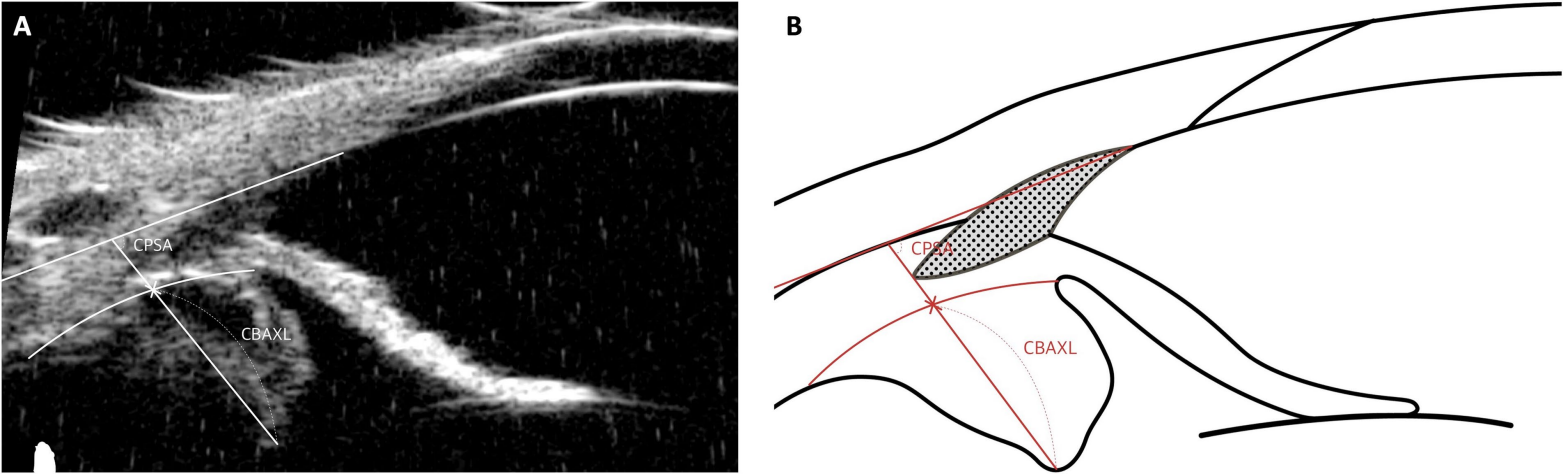
Measurement techniques of ciliary body movement parameters using UBM images in dog. This figure illustrates the methodologies used to evaluate ciliary body movement parameters. The Ciliary Body Axial Length (CBAXL) is measured by tracing a central line from the dome-shaped apex to the base of the ciliary body, accurately depicting its axial length. The Ciliary Process-Scleral Angle (CPSA), measuring the angle formed between the longitudinal axis of the ciliary body and the sclera, is essential for assessing the anatomical relationship between the ciliary body and the sclera.

The specific parameters used to evaluate ciliary body movement are as follows (Figure 7):

1. Ciliary Body Axial Length (CBAXL): This is determined by drawing a line from the apex of the dome-shaped ciliary body through its center to measure the CBAXL.
2. Ciliary Process-Scleral Angle (CPSA): This angle is measured by calculating the angle formed between the longitudinal axis of the ciliary body and the sclera.

An increase in CBAXL serves as an indirect indicator of centripetal ciliary body movement, while the posterior movement of the ciliary body process is reflected by an increase in CPSA measurements. Simply put, contraction of the ciliary body leads to an increase in CBAXL and a reduction in CPSA values (77, 78). Understanding these dynamics in ciliary body movement enhances insight into the eye’s accommodative function and related physiological processes in both human and veterinary contexts.

In the author’s opinion, these parameters are well-suited for observing changes in the ciliary body before and after surgery or following the application of topical medications. However, changes in the ciliary body associated with glaucoma cannot be explained solely by simple contraction and relaxation. Studies in humans have shown that in glaucoma, the ciliary body may undergo atrophy (82). Therefore, when comparing the ciliary bodies of glaucomatous eyes with those of normal dogs, alternative methods beyond these parameters should be considered.

### 4.5 Ciliary body muscle parameters: CBT, Lf-CMT, LRf-CMT

The ciliary body, in addition to its role in producing AH via the ciliary process, plays a key role in the uveoscleral outflow pathway (83, 84). Since this pathway passes through the interstitium of the ciliary muscle and is thought to be influenced by the contraction and relaxation of the ciliary muscle, monitoring structural changes in the ciliary musculature may be the most effective way to confirm the involvement of uveoscleral outflow (21, 22, 43, 48).

**Figure 8:**
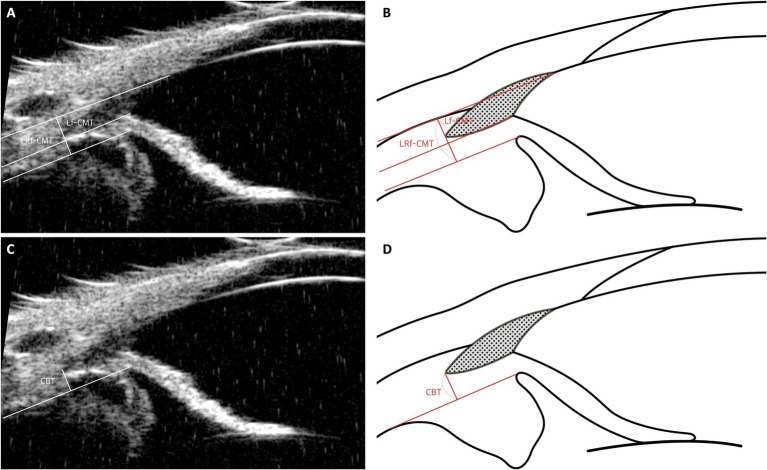
Measurement techniques of ciliary body muscle parameters using UBM images in dog. This figure presents the methods for assessing ciliary muscle parameters through UBM imaging. **(A, B)** The Lf-CMT (Longitudinal Fiber Ciliary Muscle Thickness) is determined by drawing a line through the inner scleral layer and the angle recess, with another line parallel to the inner scleral layer originating from the point where the iris root’s inner layer meets the pectinate ligament. The LRf-CMT (Lateral Root Fiber Ciliary Muscle Thickness) measurement involves tracing a line through the inner scleral layer and the angle recess, and a parallel line from the point where the outer layer of the iris root connects to the dome-shaped ciliary body. **(C, D)** The CBT (Ciliary Body Thickness) is measured as the distance from the posterior end of the ciliary cleft (CC) to the edge of the ciliary pigmented epithelium, identified by its hyperechoic appearance on UBM images.

In research conducted by Park et al., ciliary body thickness (CBT) was defined with the understanding that the attached outer portion of the ciliary body is negligible compared to the inner portion. Thus, CBT was measured as the distance between the posterior end of the CC and the edge of the ciliary pigmented epithelium, which appears as hyperechoic on UBM images (Figures 8C,D) (19). These parameters reflect changes in ciliary body thickness resulting from the relaxation and contraction of the ciliary muscle.

Building on this, our previous study identified changes in ciliary muscle fiber thickness following phacoemulsification and hypothesized that these changes may be related to the uveoscleral pathway. This led to the definition of parameters such as longitudinal fiber of ciliary muscle thickness (Lf-CMT) and longitudinal and radial fiber of ciliary muscle-choroid thickness (LRf-CMT). The measurement of Lf-CMT involves drawing a line through the inner layer of the sclera and the angle recess, with another line parallel to the inner scleral layer starting from where the inner layer of the iris root meets the PLs. The measurement of LRf-CMT requires drawing a line through the inner layer of the sclera and the angle recess, along with a parallel line starting from the point where the outer layer of the iris root meets the dome-shaped ciliary body (Figures 8A,B) (25).

The ciliary body is known to contract into a dome-shaped structure, moving in a centripetal or anterior inward direction, as observed in human studies (85). Therefore, depending on the location of the measurement, CBT may either increase or decrease even when the ciliary body contracts. Unlike in humans, dogs lack a clearly defined scleral spur, which limits the usefulness of CBT measurements in these cases. To overcome the limitations of CBT, alternative methods—such as measuring the thickness of the ciliary muscle using Lf-CMT and LRf-CMT—have been developed (25). The ciliary muscle is irregularly arranged when relaxed and becomes regularly packed upon contraction (85). From the perspective of the muscle bundle, the ciliary muscle tends to be thicker when relaxed and thinner when contracted, yielding consistent results. However, despite the advantage of providing more consistent outcomes than CBT, these methods are challenging to measure. Depending on the equipment used, the sclera might not be clearly visualized, making it difficult to establish precise measurement criteria and complicating the measurement process in dogs.

## 5 Drug-induced modifications in the iridocorneal angle and ciliary body

### 5.1 Understanding how drugs alter the structure and function of the iridocorneal angle and ciliary body

To date, research in veterinary ophthalmology has primarily focused on the CC. It is well known that changes in the width and size (expansion or contraction) of the ciliary cleft affect IOP (86). Therefore, drug responses are ultimately centered on how the CC is altered.

In the opinion of the author of this review paper, there are two main factors that affect the CC. The first is the miosis and mydriasis of the iris. It is thought that mydriasis exerts an effect by compressing the CC, thereby reducing its size, whereas miosis tends to enlarge the CC (15, 18). The second factor is the contraction and relaxation of the ciliary body. Relaxation of the ciliary body moves it in a posterior outward direction, reducing pressure on the CC; conversely, contraction moves the ciliary body in an anterior inward direction, increasing pressure on the CC (25).

For these reasons, it is necessary for studies to pay attention to pharmacological agents that act on the iris and ciliary body in relation to the CC. The iris is influenced by receptors in the iris sphincter muscle, which contains muscarinic receptors, and in the iris dilator muscle, which contains α₁-adrenergic receptors (87). In addition, the canine iris is highly populated with prostaglandin F (FP) receptors, resulting in miosis in response to drugs such as latanoprost (18).

The ciliary muscle is primarily controlled by parasympathetic innervation, which is mediated through the action of acetylcholine on muscarinic receptors. In the absence of parasympathetic stimulation, the ciliary muscle remains in a relaxed position. Upon parasympathetic stimulation, the ciliary muscle contracts (88, 89). Additionally, the ciliary muscle has well-developed prostaglandin receptors, particularly in dogs, where the prostaglandin F (FP) receptor is highly developed. This results in a strong responsiveness to drugs such as latanoprost, bimatoprost, and travoprost (90).

### 5.2 Influence of cholinergic antagonist drugs: tropicamide

Tropicamide is commonly used as a diagnostic agent to induce mydriasis due to its ability to rapidly dilate the pupil for a short duration. Research has investigated the effects of pharmaceutically induced mydriasis on intraocular pressure (IOP) in both dogs (15, 91).

A study by Thomas Dulaurent et al. evaluated structural changes associated with pupillary dilation, finding that the geometric angle formed by the plane of the iris root and the corneoscleral limbus (ICA) was larger in eyes with a dilated pupil compared to control eyes (15). This finding is consistent with earlier human studies, where mydriasis induced by three different mydriatics led to an enlargement of the geometric ICA. However, these results contradict a study by Rose et al., which reported that in noncataractous eyes, the ICA after pupillary dilation was smaller than before dilation (20). This discrepancy may be attributed to differences in measurement methodologies. In Rose et al.’s study, the ICA measurements included the most posterior portion of the CC, whereas Thomas Dulaurent et al. measured the geometric ICA formed by the iris root and the inner corneal surface (15, 20).

Moreover, the research by Thomas Dulaurent et al. demonstrated that the entry of the CC was narrower in eyes with dilated iris compared to control eyes. However, the mid-CC width and CC length did not differ between the two groups (15). These findings suggest that the CC width and functionality may be independent of the geometric angle formed by the iris and cornea. Their data indicated that mydriasis induced by topical tropicamide is associated with a constriction of the CC’s entry, while its central width and length remain unchanged. This phenomenon may be explained by the anterior displacement of the iris root during mydriasis, with the folds on the anterior surface of the iris root potentially contributing to the narrowing of the CC by contraction. However, the lack of change in the mid-CC width and CC length could be explained by the relaxation of the ciliary body. Previous studies in humans have shown that 0.5% tropicamide induces relaxation of the ciliary body (92). Based on these findings, it appears that although iris movement due to mydriasis may compress the CC, the relaxation of the ciliary body may mitigate this effect.

### 5.3 Influence of cholinergic drugs: pilocarpine

Pilocarpine, a direct-acting parasympathomimetic, is known for inducing contractions in the ciliary muscle (21). In human studies, pilocarpine has been shown to cause the ciliary muscle bundles to pull on the scleral spur, which leads to the opening of fluid pathways in the TM, thereby increasing trabecular outflow (93).

In a canine study conducted by Park et al., parameters such as AOD, CCW, and CCA were elevated in eyes administered pilocarpine (18). Although the aqueous humor pathway was enhanced in this canine study as it was in human studies, the underlying mechanism appears to differ between the two species. Pilocarpine administration induces miosis of the iris and contraction of the ciliary body. From the perspective of the CC, these actions are opposing; however, it is presumed that the iris constriction predominated, ultimately resulting in an expansion of the CC.

While the ciliary body’s response to pilocarpine—contraction—is similar in both humans and dogs, its effects on the TM and the CC appear to be contradictory. In humans, the ciliary muscle bundle attached to the scleral spur facilitates opening of the TM, whereas in dogs, the anteriorly inward movement of the ciliary body seems to narrow CC corresponding to the TM (94). In the author’s opinion, these opposing outcomes are likely due to the anatomical differences between humans and dogs.

### 5.4 Influence of prostaglandin analogs: latanoprost, tafluprost

Prostaglandin analogs are potent agents for reducing IOP, known for their effectiveness in both canine and human subjects. In humans, prostaglandin analogs have been shown to lower IOP through a dual mechanism: an immediate relaxation of the ciliary muscle, followed by a gradual restructuring of the extracellular matrix between muscle bundles, which enhances uveoscleral outflow (22).

In a canine study led by Park et al., after administering 0.005% latanoprost, the AOD and CCA values were higher in treated eyes compared to control eyes (18). In a parallel study led by Kwak et al., tafluprost, a prostaglandin FP receptor agonist, resulted in an increase in CCW (95). In both of these studies, however, although a direct relationship between the configuration of the CC and the reduction in IOP was proposed, the supporting evidence was less robust than anticipated.

The likely reason for the inability to correlate IOP with the CC in these studies is the insufficient consideration of uveoscleral outflow. In the case of prostaglandin analogs, relaxation of the ciliary body decreases the pressure exerted on the CC, thereby leading to an increase in its size. Furthermore, the relaxation of the ciliary muscle increases the interstitial space between muscle bundles, which in turn enhances the outflow of aqueous humor (96). Consequently, simply correlating the CC with IOP may not adequately capture the underlying relationship. By utilizing UBM, it is possible to evaluate the first aspect of the prostaglandin analog’s dual mechanism—that is, the changes in muscle thickness. Therefore, future studies employing parameters based on the ciliary muscle may help elucidate the relationship between these drugs and IOP more effectively.

## 6 Alterations in the iridocorneal angle and ciliary body post-cataract surgery

Following cataract surgery, particularly phacoemulsification, one concerning complication is glaucoma, which can lead to permanent vision loss and severe pain in dogs (97, 98). Another potential issue is POH, which occurs in 20–49% of dogs after phacoemulsification and IOL implantation (99). To better understand these complications, several studies have been conducted to investigate structural changes in the ICA of dogs, comparing its condition before and immediately after cataract surgery and IOL placement.

### 6.1 Parameter differences according to cataract progression stages

In our previous study., differences in CC parameters based on cataract stages were highlighted. As cataract progression advanced, metrics such as CCW, CCL, and CCA tended to increase. Specifically, CCW showed a rise in eyes with immature cataracts compared to normal eyes. CCL also exhibited a notable increase in both immature and mature cataracts relative to normal eyes, with this increase being more pronounced in mature cataracts compared to incipient ones. While CCA values were elevated in both immature and mature cataracts compared to normal and incipient cataract eyes, no differences were observed in ICA and AOD across the various stages of cataract progression (26).

In another study by the same author, it was observed that as cataract stages progressed, there was a consistent decrease in CBAXL. Significant differences were particularly noticeable between normal eyes and those with both immature and mature cataracts. This trend suggests a gradual relaxation of the ciliary body as cataracts advance. Although CPSA exhibited an increasing trend with cataract progression, this rise was not statistically significant across the various stages. Lf-CMT, however, showed a notable increase as cataracts developed, indicating a thickening of the longitudinal fibers of the ciliary muscle. These findings highlight that the axial length and muscle thickness of the ciliary body undergo measurable changes as cataracts progress (25).

The underlying rationale for these observations is as follows: as cataracts mature, there is a tendency for the ciliary muscle to enter a more relaxed state, leading to outward and backward shifts of the ciliary body (77, 78). These human findings are consistent with the our previous two studies in dogs. In dogs, as cataracts progress, the lens thickens, increasing tension on the zonular fibers, which promotes relaxation of the ciliary body. Given these movements of the ciliary body, it appears that the CC also undergoes expansion as cataracts advance (25, 26).

Pathological changes in the lens and the resulting alterations in the ciliary body are also well-documented in human studies. Research by Park et al. indicates that in cataract patients, the contractility of the ciliary body decreases but improves following cataract surgery. This was demonstrated through CBAXL measurements, where no difference was observed after pilocarpine instillation in cataract patients, but a increase was noted post-surgery. These findings are consistent with the results of the study by Park et al. (77)

### 6.2 Parameter modifications pre-and post-cataract surgery

In a study by Crumley et al., UBM was used to investigate the relationship between the pre-operative morphology of the ICA in dogs scheduled for cataract surgery and their post-operative IOPs. The researchers identified a weak correlation between pre-operative AOD and IOP measured one day after phacoemulsification (100).

Rose et al. suggested that POH is more likely to occur when the preoperative ICA exceeds 13 degrees, indicating that certain predisposing factors prior to phacoemulsification may trigger POH. However, the AOD and ICA did not show consistent changes before and after surgery, likely due to significant individual variations (20).

Our previous research revealed notable differences between the cataract group and the post-phaco group. The post-phaco group displayed smaller values for CCW, CCL, and CCA, suggesting a contraction of the CC following phacoemulsification. However, when assessing AOD and ICA, no differences were observed between the two groups, indicating that neither cataract progression nor phacoemulsification had a substantial impact on these parameters in canines (26).

In another our previous study, clear differences were observed between the cataract and post-surgery groups. The cataract group had a lower CBAXL value compared to the post-surgery group. Additionally, the CPSA value was lower in the post-surgery group than in the cataract group. The Lf-CMT value was higher in the cataract group but decreased following surgery. These findings indicate significant changes in these parameters before and after cataract surgery (25).

The rationale behind our previous findings is based on the behavior of the ciliary muscle. After phacoemulsification, the ciliary muscle contracts, causing the ciliary body to move inward and anteriorly (25, 26). These observations align with previous human studies, where cataract extraction led to reduced lens thickness, which may relieve zonular tension and allow for smoother movement of the ciliary body (77, 78).

## 7 Inter-breed differences in the canine anterior segment

In veterinary clinical practice, PACG is more common than primary open-angle glaucoma (POAG) in dogs, leading to a greater focus on the former (24). Canine PACG is characterized by the progressive narrowing and eventual collapse of the CC and ICA (101, 102). Breeds such as the American Cocker Spaniel (ACS), Basset Hound, Chow Chow, and Siberian Husky are known to be particularly predisposed to developing PACG, with the ACS showing the highest susceptibility, especially in North America (103). This increased predisposition in specific breeds has driven substantial research interest in the unique characteristics of the anterior segment in these dogs, resulting in various studies focused on this area. This breed predisposition has led to increased interest in the anatomical characteristics of the anterior segment, prompting comparative studies among breeds.

While POAG is rarely observed in clinical practice, it is most commonly reported in Beagles, whereas ACSs demonstrate a significantly higher susceptibility to PACG (49). A study led by Park et al. compared the anterior segment morphology of ACSs and Beagles, highlighting key differences in ocular dimensions (49). Despite similar overall globe sizes, the ACS exhibited a more compact anterior segment, characterized by a notably shallower anterior chamber depth and a thinner yet more anteriorly positioned lens compared to the Beagle (49). In human ophthalmology, the anterior position of the lens is considered a more critical factor than its thickness in determining the risk of angle closure. A more anteriorly positioned or protruding lens is associated with an increased likelihood of pupillary block, leading to greater iridolenticular contact and subsequent angle narrowing (104, 105). The findings from a Park’s study support this concept, demonstrating greater contact between the ACS’s posterior iris surface and the anterior lens capsule (49). This heightened iridolenticular contact exacerbates fluid entrapment at the pupillary margin, increasing resistance to aqueous humor outflow and resulting in a distinctive concave, sigmoidal-shaped iris configuration, which was more pronounced in ACSs compared to Beagles. These breed-specific anatomical differences may contribute to the higher prevalence of PACG in ACSs and warrant further investigation into their role in disease pathogenesis (49).

## 8 Discussion and future prospects

In veterinary science, UBM has emerged as an invaluable tool for the detailed examination of anterior eye segments, enabling precise study of the anatomical structures of anterior segment (10) UBM’s ability to accurately assess key components, such as the ICA and ciliary body, is particularly noteworthy (3). Despite its potential, research in the veterinary field remains in its early stages, with much of the initial work adopting parameters from human medical studies. However, there is a growing focus on understanding the differences in ICA anatomy between humans and animals, with increasing emphasis on the role of the CC in regulating AH outflow.

Several gaps in our understanding remain. A primary concern is the limited literature addressing the underlying causes of changes in the CC. Many existing studies simply report the expansion or contraction of the CC without providing substantive explanations. However, recent efforts appear to be more focused on elucidating these changes. While current knowledge acknowledges the role of ciliary muscle actions (both relaxation and contraction) and the oscillatory movements of the iris, in-depth investigations into the root causes are still lacking (15, 25). It is anticipated that future research will explore the effects of drug-induced modifications on the ciliary muscle and iris and how these changes influence the transformation of the CC.

Another significant gap is the limited focus on the unconventional outflow pathway of AH. Given UBM’s precision in measuring the ciliary body and studies suggesting a relationship between ciliary body dynamics and unconventional outflow, it is noteworthy that this crucial area remains under-researched (85, 96). Additionally, it is ironic that the ciliary body receives minimal attention in studies involving prostaglandin analogs, despite their well-documented effect on relaxing the ciliary muscle and subsequently reducing IOP.

Furthermore, research linking UBM-measurable parameters to IOP remains insufficient. Although existing studies do not show a clear correlation between parameters such as ICA or CC and IOP, this may be due to the compensatory mechanisms of IOP regulation. The eye adjusts both the production and outflow of AH in response to IOP fluctuations, often causing IOP to return to near baseline values (106). Nonetheless, a more in-depth exploration of the relationship between UBM parameters and IOP seems essential.

There is a notable lack of research utilizing UBM in feline studies. While PACG accounts for approximately 87% of all glaucoma cases in dogs, it represents only 2% of cases in cats (107–109). Additionally, unlike dogs, cats have anatomical differences that pose challenges for UBM imaging, particularly due to the difficulty in exposing the sclera beneath the eyelid. These factors likely contribute to the limited research on aqueous humor dynamics in felines using UBM. However, comparative anatomical studies in cats may provide valuable insights that could aid in the development of improved glaucoma treatment strategies for both dogs and humans.

Several areas for further research remain in the field of veterinary ophthalmology. In canine glaucoma, mydriasis is known to induce ICA crowding, leading to increased IOP (24, 49, 110). However, the precise underlying mechanism remains unclear. The authors hypothesize that pupillary dilation may cause anterior displacement of the iris, exerting pressure on the CC. To alleviate this effect, promoting ciliary body relaxation may help mitigate ICA crowding. Further investigation is required to elucidate these interactions and their implications for glaucoma management.

Beyond glaucoma research, UBM has also been widely utilized in human ophthalmology for diagnosing lens subluxation (111). In canine patients, lens subluxation may be detected during the preoperative evaluation for phacoemulsification, necessitating surgical interventions such as capsular tension ring placement or scleral fixation to ensure stable intraocular lens implantation (112). The ability of UBM to assess the positional dynamics and orientation of the lens may provide a valuable tool for diagnosing and managing lens subluxation in dogs, similar to its application in human medicine.

This study explores the application of UBM in canine ophthalmology, summarizing existing research, the authors’ perspectives, and directions for future investigations. It is hoped that this paper will serve as a valuable resource for researchers initiating studies using UBM as well as clinicians utilizing it in practice.
